# Pituitary metastasis of rhabdomyosarcoma: a case report and review of the literature

**DOI:** 10.1186/1752-1947-8-144

**Published:** 2014-05-09

**Authors:** Essadi Ismail, Lalya Issam, Mansouri Hamid

**Affiliations:** 1 Medical Oncology, Ibn Sina Military Hospital, Marrakesh, Morocco; 2 Radiation Oncology, Mohammed V Military Hospital, Rabat, Morocco

**Keywords:** Pituitary gland, Metastasis, Rhabdomyosarcoma

## Abstract

**Introduction:**

The pituitary gland is an uncommon site for metastases, in particular from rhabdomyosarcoma. Some authors have reported a recent increase in the incidence of metastases at infrequent sites, such as brain or bone, probably due to the expanded treatment options and the resulting improved survival. Treatment options are limited, but must be discussed and adapted to the patient profile.

**Case presentation:**

We report the case of a 17-year-old Arabic man, diagnosed with alveolar rhabdomyosarcoma of the left shoulder, who, after several cycles of chemotherapy, presented symptoms and signs of pituitary dysfunction. To the best of our knowledge, it is the first case described.

**Conclusions:**

Pituitary metastasis of rhabdomyosarcoma is a rare situation, which must be actively researched to have access to an optimal therapeutic approach.

## Introduction

The occurrence of pituitary metastasis is a very rare complication in oncology [1]. Data from many autopsy series state that pituitary metastases occur in 1 to 3.6 percent of patients with advanced tumors [2]. Tumors responsible for this type of extension are, in descending order: breast, lung, prostate, kidney and those of the gastrointestinal tract [3]. Some cases of lymphoma and plasmacytoma have been described in the literature [2,3]. Alveolar rhabdomyosarcoma is an aggressive tumor, with rapid and unpredictable evolutionary potential. Treatment options are limited, and the prognosis very poor [4]. We report in this manuscript the first case of metastasis of rhabdomyosarcoma to the pituitary gland.

## Case presentation

**Figure 1:**
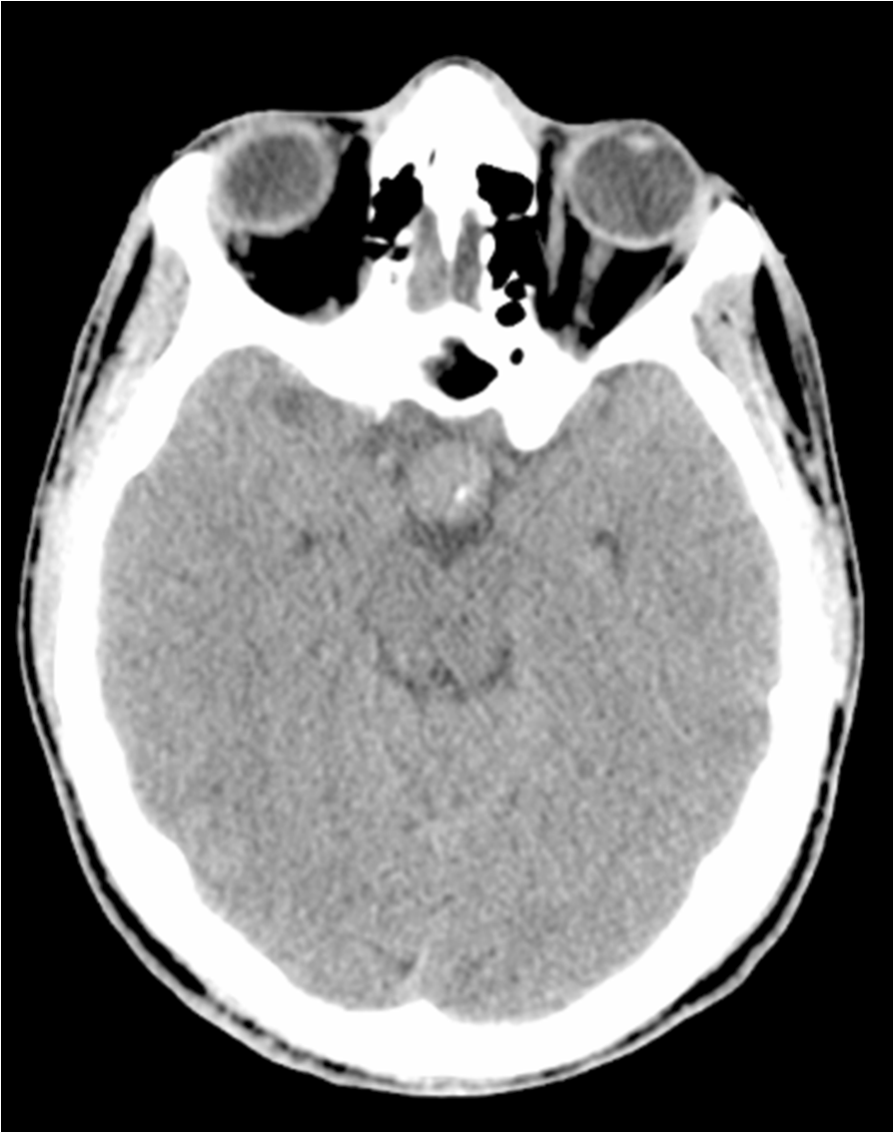
Cross-sectional computed tomography scan showing a pituitary mass.

**Figure 2:**
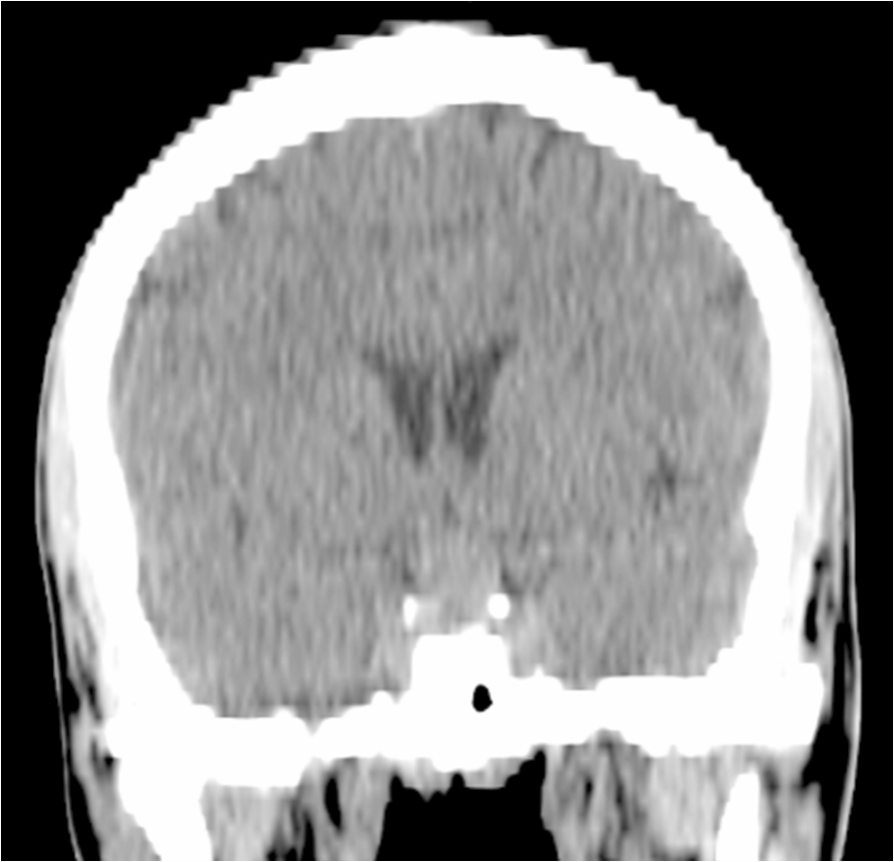
Coronal computed tomography scan section showing a pituitary mass.

**Figure 3:**
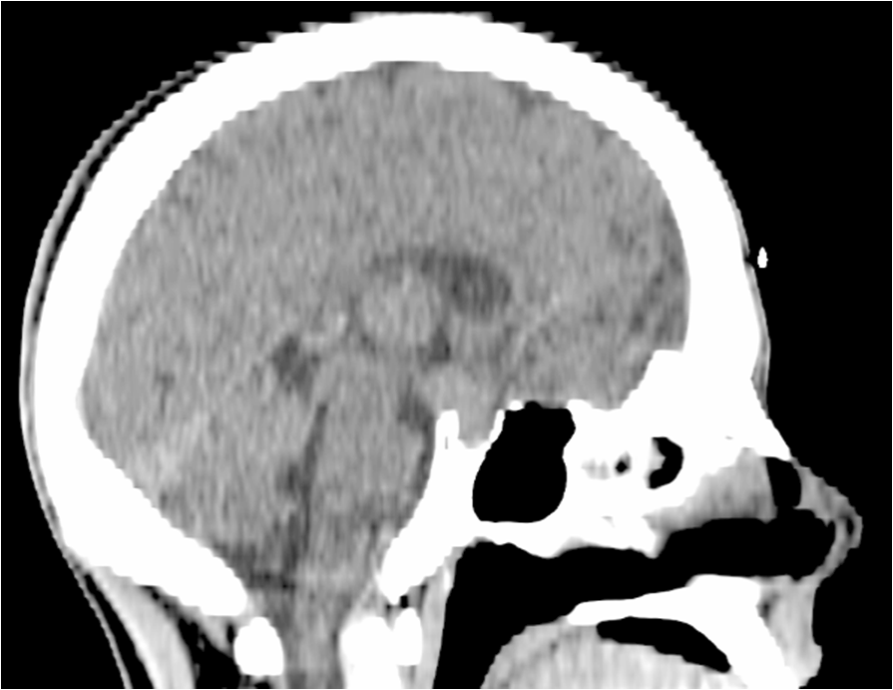
Sagittal computed tomography scan showing a pituitary mass.

**Figure 4:**
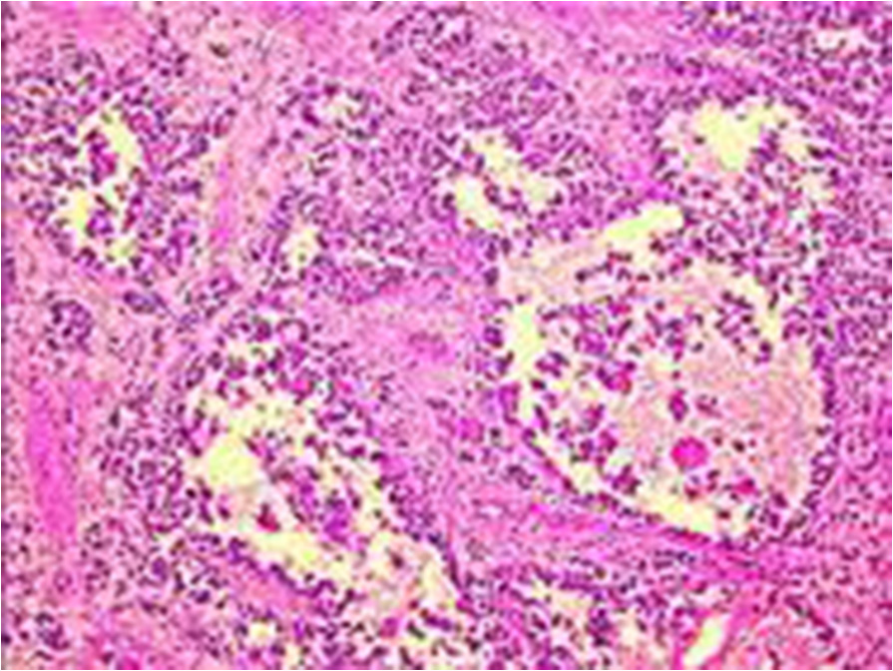
Monomorphic dense cell proliferation with a high nucleocytoplasmic ratio (alveolar rhabdomyosarcoma).

A 17-year-old Arabic man was admitted to our medical oncology department for management of alveolar rhabdomyosarcoma (RMS) of the left shoulder. The initial workup included a computed tomography (CT) scan of the body and a bone scan, which revealed lung and bone metastases. Chemotherapy with an IVA regimen was initiated (ifosfamide 5g/m2 intravenously, day 1; vincristine 2mg intravenously, day 1; doxorubicin 50mg/m2 intravenously, day 1; and zoledronic acid 4mg intravenously repeated every 21 days). His radiological and clinical evaluation after three courses of treatment showed a stable disease, with significant improvement in quality of life. After his fifth cycle of chemotherapy, he presented to our emergency department with polydipsia and polyuria, headache and vomiting associated to diplopia, and a decrease in visual acuity in his left eye. A CT scan of his brain revealed a 13 × 15mm diameter pituitary lesion (Figures 1, 2 and 3). A transsphenoidal biopsy was performed and a histopathological examination, as well as molecular diagnostics, confirmed the diagnosis of an alveolar rhabdomyosarcoma (Figure 4). He was treated by whole-brain external beam radiation therapy, at a dose of 30Gy in 10 fractions of 3Gy, using 6MV photons of linear accelerator, with spectacular regression of his neurological symptoms. He received second-line chemotherapy (gemcitabine 1000mg/m2 and oxaliplatin 100mg/m2 every two weeks); unfortunately, he died after two cycles.

## Discussion

RMS is the most common soft-tissue tumor of childhood, and responsible for approximately one-half of all soft-tissue sarcomas in this age group [5,6]. However, they are rare, representing only 3 to 4 percent of pediatric cancers overall. Approximately 350 new cases are diagnosed in the US each year, and the annual incidence in children, adolescents, and young adults under the age of 20 is 4.3 cases per one million population [5]. Lung is the most frequent site of metastases from rhabdomyosarcoma. Other sites of distant metastatic involvement include bone marrow (approximately 30 percent), bone (30 percent); omentum/ascites (16 percent), and pleura (13 percent); visceral involvement and brain metastases are rare [7-10]. To the best of our knowledge, a pituitary metastasis from rhabdomyosarcoma has never been reported in the literature. In the widest review of literature about pituitary metastases including 380 patients, none of them had a RMS as the primary localization [11]. These data confirm the authenticity of our report. The incidence of pituitary metastases on autopsy is between 1 and 3.6 percent [12,13], and if the parasellar region is included the incidence rises up to 27 percent [14]. Breast and lung carcinoma are the two most common forms of malignant tumor that metastasize to the pituitary gland. The most common symptom seems to be diabetes insipidus [15,16], reflecting a predominance of metastasis to the posterior lobe. Bilateral hemianopia is the most common type of visual field impairment [17]. Infiltration of the adjacent cavernous sinus usually induces cranial nerve III palsy, or less frequently, nerve IV palsy. Compression of nerve VI is relatively uncommon because it is well sheltered within the cavernous sinus [18]. Facial numbness due to cranial nerve V dysfunction is also rare [18]. Tumor extension to the septum pellucidum or the frontal lobes may result in cognitive deficit or psychiatric symptoms, and in anosmia if cranial nerve I is affected [16,18]. Stretching of the diaphragma sellae or ventricular distention can give rise to headaches or intracranial hypertension. All these symptoms, except for diabetes insipidus, are common in pituitary adenomas. The question is how to differentiate pituitary metastasis from pituitary adenoma in patients with a history of malignant disease, but also in those in which pituitary metastasis is the initial symptom of a malignant disease. Clinically, the presence of diabetes insipidus is very suggestive of pituitary metastasis and can be the first manifestation of a malignant neoplasm [19]. Although history or coexistence of malignancy usually leads to the diagnosis, it is of limited diagnostic value because 1.8 to 16 percent of patients with known malignancy and a sellar tumor turn out to harbor a pituitary adenoma [17,20]. With regard to neuroimaging findings, sensitive and specific criteria for differentiating pituitary metastases from pituitary adenomas have not been reported and radiological evaluation generally has not been fruitful [21], unless other metastatic brain lesions coexist. A few imaging characteristics have been reported to be helpful in differentiating pituitary metastases from pituitary adenomas; these include the following: 1) thickening of the pituitary stalk; [17,22] 2) loss of a high-intensity signal from the posterior pituitary; [23] 3) isointensity on both T1- and T2-weighted magnetic resonance images; [22] 4) invasion of the cavernous sinus; [22] and 5) sclerotic changes around the sella turcica [24]. Although these findings may indicate the possibility of metastases, they are in no way specific for pituitary metastases [2].

Treatment, mostly palliative, depends on symptoms. Surgical exploration and decompression, alone or combined with radiation, is often necessary when suprasellar extension causes progressive deterioration in vision and/or pain [25]. In our case, whole-brain external beam radiation therapy allowed improvement in symptoms, especially headache and visual field defects.

The prognosis is generally poor, because the vast majority of cases of pituitary metastasis occur in association with multiple systemic metastases and are typically associated with end-stage disease. [2]. Mean survival rates have been reported to be between 6 and 22 months, independent of the treatment strategy [17,25].

## Conclusions

Here we report the case of a patient presenting with metastatic alveolar RMS, with a large sellar mass having appeared under treatment. This metastatic event is very uncommon, making it almost impossible to perform prospective clinical trials specifically designed to compare different treatment approaches. Thus, only a greater awareness of the problem along with a more accurate and timely diagnosis, will lead to choosing the best therapies suitable to the specific patient and, in turn, improve overall prognosis.

## Consent

Written informed consent was obtained from the patient’s next-of-kin for publication of this case report and any accompanying images. A copy of the written consent is available for review by the Editor-in-Chief of this journal.

## Competing interests

The authors declare that they have no competing interests.

## Authors’ contributions

All authors read and approved the final manuscript.
